# Cross-sectional Survey on Antibiotic Prescription Practices Among Health Care Providers in Rombo District, Northern Tanzania

**DOI:** 10.24248/EAHRJ-D-18-00060

**Published:** 2018-04-01

**Authors:** Sarah R Lyimo, Geoffrey N Sigalla, Basiliana Emidi, Maseke R Mgabo, Debora C Kajeguka

**Affiliations:** a Kilimanjaro Christian Medical University College, Moshi, Tanzania; b Evangelical Lutheran Church in Tanzania, Arusha, Tanzania; c National Institute for Medical Research Headquarters, Dar es Salaam, Tanzania; d Institute of Rural Development Planning, Dodoma, Tanzania

## Abstract

**Background:**

Irrational and inappropriate antibiotic prescription is a worldwide phenomenon – increasing the threat of serious antibiotic resistance. A better understanding of health care providers' knowledge, attitudes, and prescription practices related to antibiotics is essential for formulating effective antibiotics stewardship programmes. The aim of the present study was to assess knowledge, attitudes, and prescription practices toward antibiotics among health care providers.

**Methods:**

A descriptive cross-sectional study was conducted between March and June 2017 to assess knowledge, attitudes, and prescription practices toward antibiotics among health care providers in the Rombo district of northern Tanzania. A total of 217 health care providers were interviewed using a structured questionnaire.

**Results:**

Over half of health care providers (n=111, 51.2%) strongly agreed that the inappropriate prescription of antibiotics puts patients at risk. More than half (n=112, 51.6%) reported that their decision to start antibiotic therapy was influenced by a patient's clinical condition, while 110 (50.7%) reported they were influenced by positive microbiological results in symptomatic patients. Almost two-thirds of the health care providers (n=136, 62.7%) reported that they had access to and used antibiotic therapy guidelines. Less than a quarter (n=52, 24.0%) received regular training and education in antibiotic prescription practice in their work place.

**Conclusion:**

Knowledge and prescription practice of antibiotics among health care providers was generally unsatisfactory. Training and education for health care providers is needed in the area of prescribing antibiotics.

## INTRODUCTION

Antibiotics are the most commonly prescribed drugs in clinical practice. In most parts of the world, antibiotic prescription practice and use is either irrational or inappropriate – increasing the threat of serious antibiotic resistance.^1,2^ In 2011, the World Health Organization (WHO) estimated that half of all medications are irrationally prescribed or sold worldwide.^3^ The irrational prescription of medicines is a phenomenon that prevails across both developing and developed countries.^3,4^ In African countries, for example, a systematic review of 43 studies on prescribing indicators at lower-level health care facilities indicated that the median number of medicines prescribed to clients for every encounter with a health provider was 3.1.^5^ This recent review of studies published from 1995 to 2015, included 6 studies from Tanzania, and nearly half of all encounters ended with the prescription of an antibiotic.

In Tanzania, studies have indicated that irrational prescription patterns of antibiotics by health care providers is a challenge^6,7^ and the magnitude varies widely. A recently published cross-sectional study assessed the rational prescription of medicines in 4 regions of Tanzania by studying 2,067 prescriptions from 67 health care facilities.^6^ The study revealed that more than two-thirds (67.7%) of the prescriptions were antibiotics, when compared to the optimal level of 30%, indicating that overprescribing antibiotics is a prevailing behaviour among health care providers. More alarming results were reported by an earlier study in the Northern Zone of Tanzania among 384 children – aged 1 month to 5 years being managed for acute watery diarrhoea – where 84.9% were given antibiotics.^7^ Since most acute watery diarrhoea in that region is caused by viruses, antibiotics should not have been prescribed. The results of these studies, conducted in the same region where the present study was conducted, underscore a need to assess the levels of knowledge and attitudes of the health care providers regarding prescription practices of antibiotics.

Poor knowledge about antibiotics may be a factor influencing prescribers' attitudes toward antibiotic use and prescription practices, which then becomes a problem for patients and the community.^8^ Lack of confidence and training about the rational use of antibiotics among prescribers and dispensers is a serious problem, especially when antibiotics are prescribed in the absence of cause of illness, thus increasing the risk of antibiotic resistance and adverse reactions.^9–11^ Several factors contribute to the inappropriate prescription of antibiotics, including a health care provider's knowledge and experiences, uncertain diagnosis, patient expectations, pharmaceutical marketing influences, and unregulated antibiotic dispensing.^12^ Additional factors, such as inappropriate antibiotic use in agriculture and food-producing animals, also pose a significant threat to human and animal drug resistance. The challenge of the latter is that there is no control of antibiotic use in agriculture and food-producing animals in Tanzania.^13^ To that end, there is a risk of humans acquiring resistant pathogens from food-producing animals – such risk must be examined and reduced. The consequences of irrational prescriptions of antibiotics are immense: the practice is associated with immediate economic consequences to the user^4^ and the overuse of antibiotics is a cause of bacterial drug resistance, which is increasingly becoming an important global public health problem.^14^

Understanding the prescribing pattern of antibiotics in Tanzania is important, considering that prescriptions in the country are frequently’ based on clinical presentation of client's medical conditions. Due to an increasing number of patients and the limitations related to the nationwide availability of reliable laboratory services for all conditions, especially in hard-to-reach rural areas, health care providers tend to base their diagnoses on clinical judgment, while basing their decisions for treatment on the national *Standard Treatment Guidelines and Essential Medicines List* and other related guidelines produced by the Ministry of Health and Social Welfare.^15^ Studies at the local level are important to narrow the knowledge gap regarding antibiotic prescription practices among health care providers. A better understanding of physicians' knowledge, attitudes, and practices toward prescribing antibiotics is essential for formulating effective antibiotic stewardship programmes.^16^ Therefore, the objective of the present study was to assess knowledge, attitudes, and prescription practices related to antibiotics among health care providers in the Rombo district of northern Tanzania.

## METHODS

### Setting, Design, and Population Sampling

This descriptive study was conducted from March to June 2017, in Rombo District, Kilimanjaro, Tanzania. Kilimanjaro has 7 districts: Hai, Moshi rural, Moshi urban, Mwanga, Rombo, Same, and Siha. Rombo is bordered to the north and east by Kenya, to the west by the Hai and Siha districts, and to the south by the Moshi rural district. Within the Rombo district is a total of 43 operating health facilities, including 37 dispensaries, 4 heath centres, and 2 hospitals, and 326 health care providers.^17^ The Rombo district was randomly selected using the rotary method: each district name was written in a piece of paper, then 1 paper was independently selected. Systematic random sampling was employed to select prospective respondents: a starting point was chosen at random from a list of all health care providers and choices, thereafter, were made at regular intervals. The study included health care providers employed in government and non-government health facilities working in the facility for more than 1 year. We excluded providers in the internship programmes. The study was conducted among 217 health care providers from government and non-government health facilities.

### Sample Size Calculation

The minimum sample size was estimated using a formula expressed as N = [Z^2^ P(1 P)]/(d^2^), where Z is the value (1.96 for 95% confidence interval [CI]), P represents prevalence (0.846), and d is the minimum tolerable error at 95% CI, expressed as a decimal (0.05). The prevalence of antimicrobial prescription and dispensing was 84.6%.^20^ Therefore, N = 200 + 10% (of 200) = 220, with 10% repre senting the non-response correction.

### Questionnaire

Data were collected using a structured, anonymous, and self-administered 4-part questionnaire. The questionnaire content was based on a survey described in an Indian study,^16^ but adopted to Tanzania and modified for the purposes of this study. The questionnaire was validated using a small group of medical residents before it was distributed among the target population. Based on the pilot study, the questionnaire was modified and improved on advice of relevant experts in statistics and epidemiology. The final version of the questionnaire had 16 key questions subdivided into 3 categories: knowledge and attitudes, prescription practices, and accessibility and use of antibiotic therapy guidelines.

Briefly, part 1 recorded sociodemographic and health facility characteristics. Part 2 was composed of 8 questions on knowledge and attitudes of antibiotic prescription practices: ‘Does inappropriate antibiotic prescribing put patients at risk?’, ‘Is it always better to overprescribe antibiotics than underprescribe?’, ‘Should everyone be able to buy antibiotics without a prescription?’, ‘Is antibiotic resistance a problem in my daily practice?’, ‘Is antibiotic resistance a significant problem in my health facility?’, ‘Antibiotic resistance is a significant worldwide problem?’, ‘Are infectious diseases services at my hospital easily accessible?’, and ‘Are infectious diseases services at my health facility very helpful?’. Each question in this part was assessed using a 5-point Likert scale ranging from strongly agree to strongly disagree.

Part 3 had 5 questions about prescription practice: ‘Which of these factors may influence your decision to start antibiotic therapy?’, ‘Do you ever try to make sure that your antibiotic prescribing is cost effective?’, ‘Which of these do you think are important causes of inappropriate antibiotic use?’, ‘Which of the following do you think may help control antibiotic resistance?’, and ‘Have you received regular training and education in antibiotic prescribing in your work place?’.

Part 4 had 3 questions on the accessibility and use of antibiotic therapy guidelines: ‘Does your health facility provide guidelines for diagnosis and management of patient with infective problem?’, ‘How accessible are these guidelines?’, and ‘Do you follow the recommendations of your health facility antibiotic guidelines?’.

### Data Analysis

Data were analysed using IBM SPSS Statistics for Windows, Version 22.0 (IBM Corp, Armonk, NY, USA). Data analysis was conducting using descriptive statistics, including frequencies and percentages.

### Ethics Approval and Consent to Participate

Permission to conduct the study was gained from the Kilimanjaro Christian Medical University College Research Ethics Review Committee and from the district medical officer. Written informed consent was obtained from all participants who voluntarily agreed to take part in this study.

## RESULTS

### Demographic Characteristics

The response rate was 98.6%. Out of the 217 participants, 140 (64.5%) were female. A high proportion of participants (n=89, 41.0%) were under 30 years, followed by those between the ages of 31 and 40 years (n=78, 35.9%) and those over 40 years (n=50, 23.1%). Over two-fifths (n=94, 43.3%) of the participants worked at a dispensary. A majority of health care providers (n=149, 68.7%) were assistant nursing officers, followed by clinical officers (n=46, 21.2%), assistant medical officers (n=10, 4.6%), medical doctors (n=6, 2.8%), and nursing officers (n=6, 2.8%). Regarding years of experience, nearly a quarter (n=53, 24.4%) of the health care providers had more than 10 years of work experience and almost half (n=106, 48.8%) had between 3 and 5 years of work experience. **Table 1** summarizes the complete sociodemographic characteristics of the study participants.

**TABLE 1.** Demographic Characteristics of the Study Population in Rombo District (N=217)

Variable	n (%)
Gender	
Male	77 (35.5)
Female	140 (64.5)
Age (in years)	
Less than 30	89 (41.0)
31 to 40	78 (35.9)
More than 40	50 (23.1)
Type of health facility	
Hospital	53 (24.4)
Health centre	70 (32.3)
Dispensary	94 (43.3)
Medical role/cadre	
Assistant nursing officer	149 (68.7)
Nursing officer	6 (2.8)
Assistant medical officer	10 (4.5)
Clinical officer	46 (21.2)
Medical doctor	6 (2.8)
Years of experience	
Less than 2 years	16 (7.4)
3 to 5 years	106 (48.8)
6 to 10 years	42 (19.4)
More than 10 years	53 (24.4)

### Knowledge and Attitudes Toward Antibiotics Among Health Care Providers

**FIGURE 1.**
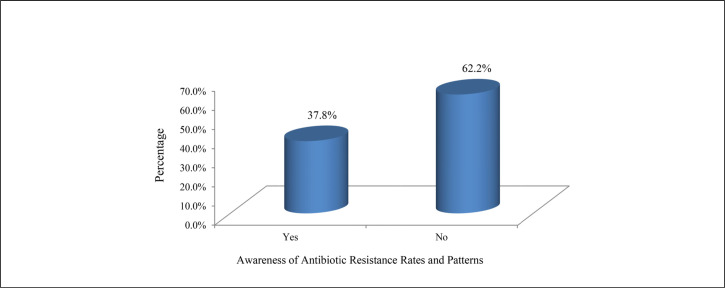
Awareness of Antibiotic Resistance Rates and Patterns

Over half (n=111, 51.2%) of the participants strongly agreed and almost all of the others (n=97, 44.7%) agreed that inappropriate prescription of antibiotics puts patients at risk. A majority of health care providers either disagreed (n=81, 37.3%) or strongly disagreed (n=65, 30.0%) that overpre-scribing antibiotics is always better than underprescribing. More than two-fifths (n=90, 41.5%) disagreed that everyone should be able to buy antibiotics without prescription, and more than half (n=132, 60.8%) agreed that antibiotic resistance was a worldwide problem. A majority of participants agreed that infectious disease services are easily accessible (n=178, 82.0%) and that infectious disease services are helpful (n=172, 79.3%). **Table 2** presents participant knowledge and attitudes on antibiotics. Only 82 (37.8%) health care providers were aware of antibiotic resistance rates (**Figure 1**).

**TABLE 2.** Knowledge and Attitude on Antibiotics Among Healthcare Providers in Rombo District (N=217)

	Response n (%)				
Question	Strongly Agree	Agree	Neutral	Disagree	Strongly Disagree
Does inappropriate antibiotic prescribing put patients at risk?	111 (51.2)	97 (44.7)	0 (0.0)	7 (3.2)	2 (0.9)
Is it always better to overprescribe antibiotics than to underprescribe?	13 (6.0)	23 (10.6)	35 (16.1)	81 (37.3)	65 (30.0)
Should everyone be able to buy antibiotics without a prescription?	6 (2.8)	24 (11.1)	16 (7.4)	90 (41.5)	81 (37.4)
Is antibiotic resistance a problem in my daily practice?	4 (1.8)	107 (49.3)	1 (0.5)	92 (42.4)	11 (5.1)
Is antibiotic resistance a significant problem in my health facility?	2 (0.9)	100 (46.1)	7 (3.2)	104 (47.9)	4 (1.8)
Is antibiotic resistance is a significant worldwide problem?	26 (12.0)	132 (60.8)	2 (0.9)	57 (26.3)	0 (0.0)
Are infectious disease services at my hospital easily accessible?	19 (8.8)	178 (82.0)	0 (0.0)	19 (8.8)	1 (0.5)
Are infectious disease services at my health facility very helpful?	21 (9.7)	172 (79.3)	9 (4.1)	13 (6.0)	2 (0.9)

### Health Care Provider Practices on Antibiotic Prescriptions

**FIGURE 2.**
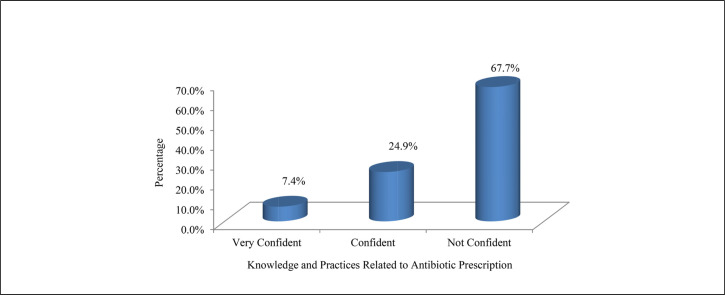
Confidence About Knowledge and Practices Related to Antibiotic Prescribing

More than half (n=112, 51.6%) of the health care providers reported that their decision to start antibiotic therapy was influenced by patient's clinical condition, while about half (n=110, 50.7%) indicated they were influenced by positive microbiological results in symptomatic patients. Almost two-fifths (n=85, 39.2%) responded that most of the time they try to make sure that the antibiotic prescription is cost effective. Of the important causes of inappropriate antibiotic use, poor skills and knowledge (n=140, 56.5%) were identified as the leading cause, followed by lack of interest in the subject of antibiotic prescribing and infection management (n=63, 29.0%). On important strategies to control antibiotic resistance, 119 (54.8%) were in favour of providing education to physicians on appropriate antibiotic therapy, while 70 (32.3%) suggested including knowledge of pathogens and antibiotic susceptibility results. Only 52 (24.0%) received regular training and education in antibiotic prescription in their work place (**Table 3**). Less than one-tenth (n=16, 7.4%) of health care providers were ‘very confident’ in their knowledge and practices on antibiotic prescription (**Figure 2**).

**TABLE 3.** Responses from Healthcare Providers on Their Antibiotic Prescription Practices (N=217)

Questions	Potential Answers	n (%)
Which of these factors may influence your decision to start antibiotic therapy?	Patient's clinical condition	112 (51.6)
	Positive microbiological results in symptomatic patients	110 (50.7)
	Wanting to satisfy the senior treating physician	0 (0.0)
	Worry of missing patients with possible infections	3 (1.4)
Do you ever try to make sure that your antibiotic prescribing is cost effective?	Always	51 (23.5)
	Most of the time	85 (39.2)
	Never	14 (6.5)
	Rarely	15 (6.9)
	Sometimes	52 (24.0)
Which of these do you think are important causes of inappropriate use of antibiotic?	Poor skills and knowledge	140 (56.5)
	Unrestricted availability of antibiotics	16 (7.4)
	Inadequate supervision	10 (4.6)
	Lack of interest in the subject of antibiotic prescribing and infection management	63 (29.0)
	Lack of effective hospital policies	2 (0.9)
	Overworked health care personnel	9 (4.1)
Which of the following do you think may help control antibiotic resistance?	Treating infection, not contamination or colonization	22 (10.1)
	Physician education on appropriate antibiotic therapy	119 (54.8)
	Consulting with infectious diseases experts	4 (1.8)
	Providing local antibiotic guidelines	8 (3.7)
	Knowledge of pathogens and antibiotic susceptibility test results	70 (32.3)
	Obtaining local antibiotic resistance profile	4 (1.8)
Have you received regular training and education in antibiotic prescribing in your work place?	Yes	52 (24.0)
	No	165 (76.0)

### Accessibility and Use of Antibiotic Therapy Guidelines

A majority (n=136, 62.7%) of health care providers reported that antibiotic therapy guidelines were accessible and used. About one-third (n=73, 33.6%) reported that the guidelines provided were comprehensive, while half (n=109, 50.2%) indicated that the guidelines were not always accessible when needed. Almost a quarter (n=48, 22.1%) of health care providers reported they never follow the recommendations of the antibiotic guidelines, while more than a quarter (n=63, 29.0%) reported they always follow the antibiotic guidelines (**Table 4**).

**TABLE 4.** Accessibility and Use of Antibiotic Therapy Guidelines (N=217)

Question	Potential Answers	n (%)
Does your health facility provide guidelines for diagnosis and management of patient with infective problem?	Yes, but limited	136 (62.7)
	Yes, but not helpful	6 (2.8)
	Yes, comprehensive	73 (33.6)
	I do not know	2 (0.9)
How accessible are these guidelines?	Limited access/access with difficulty	109 (50.2)
	Widely accessible	99 (45.6)
	I do not know	9 (4.1)
Do you follow the recommendations of your health facility antibiotic guidelines?	Never	48 (22.1)
	Rarely	16 (7.4)
	Sometimes	43 (19.8)
	Most of the time	47 (21.7)
	Always	63 (29.0)

## DISCUSSION

This study assessed knowledge, attitudes, and prescription practices related to antibiotics among health care providers in the Rombo district in northern Tanzania. No previous study in this area focused on prescribing practices among health care providers. Health care providers play an important role in antibiotic misuse and, thereafter, the development of antibiotic resistance. This is due to either lacking knowledge about appropriate antibiotic prescription practices or being reluctant to practice caution.

### Knowledge and Attitudes of Antibiotic Use Among Health Care Providers

In 2006, a study from the same district documented that antibiotic prescriptions should only be made if health care providers have adequate knowledge of the patient's health and are satisfied that they are serving the patient's needs.^18^ In this study, health care providers reported that inappropriate prescription of antibiotics puts patients at risk. However, in our study, a small proportion of health care providers did not agree with these statements. The same result has also been reported in Saudi Arabia.^16^ A study conducted in Ghana reported that 69.1% of health care providers agreed that inappropriate antibiotic use might lead to dangerous allergies, which could cause death.^19^ Another study conducted by Tegagn et al reported that inappropriate antibiotic use can lead to resistance, treatment failure, increased adverse effects, and an additional burden of medical cost to the patient.^20^ Furthermore, a study by Kumar et al stated that inappropriate antibiotic prescribing results in a 5-fold mortality increase in patients.^21^ Our study suggests that there is a lack of knowledge on the effect of inappropriate prescription of antibiotics. Moreover, in this study, a small proportion of health care providers agreed on the statement ‘it is better to overprescribe than underprescribe’. Similar observations have been reported elsewhere.^16^

In this study, half of the health care providers disagreed that antibiotic resistance is a significant problem in their respective health facilities, while 38.7% were unaware of antibiotic resistance rates and patterns. This may be due to inadequate surveillance of antibiotic resistance in Tanzania. The same has been reported by Baadani et al.^16^ However, several studies have reported antimicrobial resistance in the region.^22,23^

Researchers from Saudi Arabia have suggested that surveillance systems of antimicrobial usage and resistance should include efforts to ensure timely dissemination of information to all health care providers and stakeholders.^16^

### Antibiotic Prescription Practices Among Health Care Providers

Several factors, such as clinical conditions and positive microbiological tests in symptomatic patients have been reported to influence provider decisions to start antibiotic therapy.^16^ This is encouraging in environments where laboratory facilities and effective hospital policies are available. However, a supportive environment is only 1 of several factors that influence antibiotic prescribing behaviours. Poor skills and knowledge are the leading causes of inappropriate antibiotic prescribing and use. In this study, a lack of interest in the subject of antibiotic prescription and infection management was identified as a cause of inappropriate antibiotic use by 29.0% of participants. This is a higher proportion than that reported by Baadani et al, who reported that only 6.6% of health care providers believed that lack of interest in the subject of antibiotic prescription and infection control was the cause of inappropriate antibiotic use.^16^

On important strategies to control antibiotic resistance, more health care providers were in favour of providing education to physicians on appropriate antibiotic therapy, while others suggested consideration of knowledge of pathogens and antibiotic susceptibility results. Furthermore, 24.0% reported that they did not receive regular training and education in antibiotic prescription. This is nearly the same as the study by Baadani et al, which reported that 34.9% of respondents did not receive regular training.^16^ As suggested, there is an urgent need for carefully planned education and training programmes to address the knowledge gaps and support appropriate evidence-based antimicrobial prescribing practices among health care providers.^16^

### Accessibility and Use of Antibiotic Therapy Guidelines

This study revealed that access to clinical or treatment guidelines was often limited or nonexistent. This may explain why some health care providers reported they never follow antibiotic guideline recommendations, which could provide them with information on how to safely prescribe antibiotics. Instead, by ignoring recommendations, they continue inappropriate prescription practices and, hence, contribute to increasing antibiotic resistance rates.

As part of a global effort to fight against the development of antibiotic resistance, the Global Antibiotic Resistance Partnership (GARP)–Tanzania aimed to develop policy recommendations to govern the appropriate use of antibiotics. Identified priority areas include, among others, improving hospital practices; rationalising antibiotic use in the community; educating health professionals, policymakers, and the public on sustainable antibiotic use; and ensuring political commitment to meet the threat of antibiotic resistance. However, in Tanzania, there are no current national or local data on antimicrobial surveillance. Moreover, in addition to the recommendations made by GARP, more reports are needed from the local and national levels on surveillance of antibiotic prescribing and resistance.^24^ The National Centre for Adverse Drug Reactions monitoring, under the Tanzania Food and Drugs Authority, aims to analyse and disseminate information needed to support drug prescribing and regulation strengthening.

### Study Strengths and Limitations

Although this is the first report on antibiotic prescribing among providers in the Rombo district, and probably in the Kilimanjaro region, we feel that the findings are of generalized interest. This study did not interview pharmacists or patients; adding their perspectives could have provided a richer picture of prescription practices and antibiotic use. The study also did not explore the commonly used antibiotics in the study area and did not try to achieve a balance between provider location and level of practice.

## CONCLUSION

In this study, health care provider knowledge and prescription practices related to antibiotics were generally unsatisfactory. Our research showed that there is a clear need for training and education for health care providers in the area of antibiotic prescribing, and that antibiotic therapy guidelines should be easily accessible and effectively used.
